# Global clinical landscape and multidisciplinary translational frontiers of FLASH radiotherapy: a systematic review and visualized bibliometric mapping based on WoSCC and PubMed (2014–2025)

**DOI:** 10.3389/fmed.2026.1864006

**Published:** 2026-07-29

**Authors:** Yupeng Di, Chen Liu, Yingjie Wang, Boning Cai, Lingling Meng

**Affiliations:** 1 Department of Radiation Oncology, Air Force Medical Center, Fourth Military Medical University, Beijing, China; 2 Department of Radiation Protection Medicine, School of Preventive Medicine, Fourth Military Medical University, Xi’an, China; 3 Ministry of Education Key Lab of Hazard Assessment and Control in Special Operational Environment, Xi'an, China; 4 Department of Radiation Oncology, Senior Department of Oncology, The First Medical Center of PLA General Hospital, Beijing, China

**Keywords:** bibliometric analysis, clinical translation, dosimetry, FLASH radiotherapy, radiation oncology, ultra-high dose rate

## Abstract

**Background:**

Ultra-high dose rate FLASH radiotherapy (FLASH-RT) holds the potential to revolutionize oncology by dramatically sparing normal tissue while maintaining tumor control. To capture both physical breakthroughs and biomedical translation, this study provides a comprehensive bibliometric mapping to evaluate the global research landscape and translational frontiers of FLASH-RT.

**Methods:**

To ensure maximum comprehensiveness, a systematic dual-database retrieval was conducted using the Web of Science Core Collection (WoSCC) and PubMed for articles published between January 1, 2014, and December 31, 2025. Following software-assisted deduplication using bibliometrix and independent, researcher-guided manual screening to exclude non-relevant document types, analytical protocols utilizing the R package bibliometrix, VOSviewer, and CiteSpace were applied to visualize publication trends, co-authorship networks, and keyword bursts.

**Results:**

A final analytical cohort of 807 high-quality articles was identified, revealing a rapidly accelerating, non-linear growth pattern relative to the 2014 baseline, with a marked surge after 2021, corresponding to the onset of prospective human FLASH-RT trials. The fitted annual-output trend followed a quadratic polynomial model with R^2^ = 0.9458. The United States (30.4%) and China (18.7%) led quantitatively, while Switzerland drove deep multi-institutional innovation. We identified a definitive interdisciplinary paradigm shift: early investigations focused on fundamental physics and X-ray dosimetry, whereas contemporary research has rapidly pivoted toward proton FLASH therapy, oxygen depletion kinetics, and phase-1 clinical workflows. Furthermore, keyword burst detection highlighted “ion recombination” and “delivery methods” as the most critical ongoing technical challenges.

**Conclusion:**

By integrating extensive data from both WoSCC and PubMed, this study documents the safety-led maturation of FLASH-RT from an experimental physics curiosity to a viable clinical precision tool. The evolving synergy between dose-rate optimization and clinical oncology emphasizes that solving real-time dosimetric challenges remains the essential frontier for achieving widespread clinical adoption and widening the therapeutic window.

## 1 Introduction

FLASH radiotherapy (FLASH-RT), an innovative modality, has emerged as one of the most promising frontiers in radiation oncology over the last decade. By administering radiation at ultra-high dose rates (equal to or greater than 40 Gy/s)—several orders of magnitude higher than conventional radiotherapy (3–10 Gy/min)—FLASH-RT leverages the “FLASH effect” to demonstrate a significant reduction in normal tissue complication probability (NTCP) while preserving equivalent tumor control (1–3). This crucial therapeutic advantage expands the therapeutic window into a “high efficacy, low toxicity” dimension, positioning FLASH-RT as a potentially disruptive technology for cancer treatment (4–8). Recent milestones, such as the inaugural FAST-01 and FAST-02 human trials, have catalyzed a shift in the field from fundamental physics verification toward complex clinical translation and the prospective integration of FLASH-RT into clinical protocols for targeted radical treatments and the management of complex metastases, although current trials are primarily restricted to establishing feasibility, safety, and non-inferiority in palliative cohorts (9–12).

Despite this burgeoning interest, the rapid evolution of the field has created a knowledge gap regarding the shifting research paradigms. Early investigations were primarily centered on the engineering of high-output accelerators and basic dosimetric characterization. However, as the field matures, the focus is increasingly aligning with molecular biology, real-time beam monitoring, and clinical workflows. Understanding these dynamic transitions is essential for guiding future research priorities and optimizing clinical trial designs.

Bibliometric analysis, a robust quantitative methodology, provides a rigorous framework for evaluating the evolution of a scientific field (13, 14). By analyzing citation patterns, collaborative networks, and keyword trajectories, this approach identifies influential contributors and emerging “hotspots” that traditional reviews might overlook. While some bibliometric studies have previously mapped the early stages of FLASH research (15, 16), they have largely relied on single-database retrievals. The recent explosion of interdisciplinary clinical data and the introduction of proton-based FLASH therapy necessitate a more comprehensive and nuanced analysis utilizing dual-database integration to fully capture both physical breakthroughs and biomedical progress.

This study aims to deliver a comprehensive bibliometric evaluation of the global FLASH-RT landscape from 2014 to 2025 by systematically integrating data from the two most authoritative databases: the Web of Science Core Collection (WoSCC) and PubMed. Beyond simple descriptive statistics, this research investigates the maturation of the field, the transition from preclinical models to mainstream clinical implementation, and the emerging interdisciplinary synergies between physics, radiobiology, and clinical radiation oncology. These findings are intended to offer critical evidence-based insights for clinicians and researchers, fostering more focused investigations and accelerating the integration of FLASH radiation into standard oncological care.

This study is designed as a cross-sectional bibliometric mapping, capturing a global retrospective database snapshot of all peer-reviewed publications within a fixed census window (2014–2025), which has been conducted and reported in compliance with the STROBE (Strengthening the Reporting of Observational Studies in Epidemiology) guidelines (17). Furthermore, to ensure academic transparency, we confirm that this manuscript was generated, analyzed, and written without the use of Artificial Intelligence technologies.

## 2 Materials and methods

### 2.1 Search strategy and dataset selection

In this cross-sectional bibliometric study, the systematic search for eligible publications was executed on a single day (December 31, 2025). This study is defined as cross-sectional because it provides a comprehensive academic snapshot of the literature indexed in the databases as of a specific census date (December 31, 2025). To ensure maximum comprehensiveness and capture both the physical engineering breakthroughs and the biomedical/clinical translations of FLASH-RT, two highly authoritative databases were selected: the Web of Science Core Collection (WoSCC) and PubMed. This dual-database integration strategy was implemented to minimize single-database bias, combining WoSCC’s robust citation mapping capabilities with PubMed’s extensive coverage of clinical and biomedical literature.

The search strategies were rigorously tailored to the syntax of each database: the WoSCC search strategy was TS = (“flash radiation oncology” OR “flash radiotherapy” OR “flash effect” OR “flash-effect” OR “flash radiation therapy” OR “flash irradiation” OR “flash-radiotherapy” OR “flash rad*”), and the PubMed search strategy employed was (“flash radiation oncology” OR “flash radiotherapy” OR “flash effect” OR “flash-effect” OR “flash radiation therapy” OR “flash irradiation” OR “flash-radiotherapy” OR “flash rad*”). We opted to search for terms with and without hyphens to comprehensively capture spelling variations across different editorial formats. Standalone technology-based terms such as “ultra-high dose rate” or “UHDR” were intentionally excluded from the primary keyword query because exploratory searches showed they retrieved thousands of unrelated papers from high-speed fluid dynamics, laser chemistry, and aerospace engineering. Instead, these technology-focused publications were captured only when co-indexed with the core “FLASH” terminology, thereby maintaining specificity for FLASH radiotherapy while reducing irrelevant retrieval from unrelated engineering fields.

**Figure 1:**
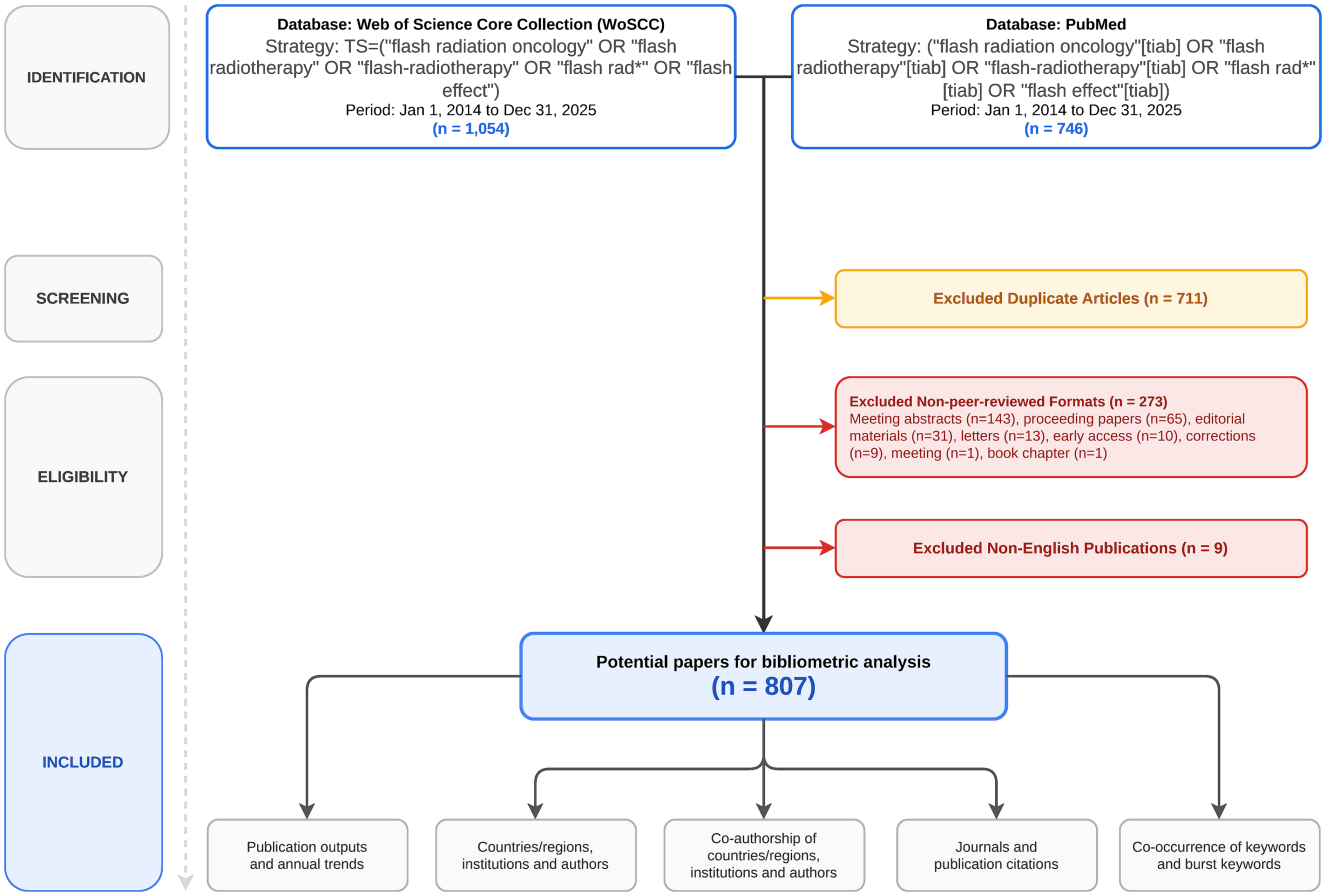
PRISMA flow diagram of the literature selection process. The flowchart illustrates the systematic workflow of article identification, screening, eligibility evaluation, and final inclusion. A total of *n* = 1,054 records from the Web of Science Core Collection (WoSCC) and *n* = 746 records from PubMed were initially identified. After removing duplicates (*n* = 711), non-peer-reviewed formats (*n* = 273, including meeting abstracts, letters, editorial materials, and corrections), and non-English publications (*n* = 9), a cohort of *n* = 807 publications was selected for targeted bibliometric mapping across five principal analysis domains (annual trends, spatial collaborations, co-authorship networks, journal overlays, and burst keywords).

The screening process followed a combined automated and manual pipeline to ensure data integrity. Initial deduplication of overlapping records between the databases was performed automatically using the R package bibliometrix. Subsequently, to ensure academic quality and exclude non-relevant document types, two researchers independently screened the titles and abstracts of the unique records. Evolving disputes were resolved through consensus with a third reviewer. The exclusion criteria were strictly applied to non-peer-reviewed or secondary document types, including meeting abstracts, proceeding papers, editorial materials, letters, early access articles, correction notices, meeting records, and book chapters. To maintain scientific rigor, when corrections were identified, the erratum/correction notice itself was excluded as a document type, but the original corrected research paper was fully retained and updated with the modified metadata to prevent data duplication. Only publications written in English were included to maintain textual standardization. The detailed selection flow and screening logic are illustrated in the PRISMA 2020 flow diagram (Figure 1).

To address the risk of bias within this bibliometric framework, we assessed potential datasets for publication bias and citation inflation. This was accomplished by cross-verifying citation counts across both databases and comparing publication distributions against established journal impact factors and author impact metrics (h-index and g-index). This process ensured that the quantitative prominence of the included nodes reflected objective scientific influence rather than indexing anomalies.

### 2.2 Data analysis and visualization

The raw data from both databases were harmonized and exported in plain text formats compatible with bibliometric software. The R programming language (v4.4.1) (18) and the R package bibliometrix (v5.0) (19) were used to quantify and perform descriptive statistics on the number of publications, top journals, and local citation impacts. VOSviewer (v1.6.17) (20) was employed to generate visual network maps by calculating the co-occurrence of and connections among authors, institutions, and keywords. The R package circlize (v0.4.15) (21) was utilized to construct the visualization of international country cooperation networks. Furthermore, CiteSpace (v6.1. R6) (22) was utilized to identify citation bursts in keywords and generate dual-map overlays, which track the interdisciplinary flow of knowledge. Journal impact factors (IF) were sourced from the 2024 Journal Citation Reports (JCR).

To standardise the interpretation of the network maps, we define the key bibliometric metrics as follows: “Link strength” represents the frequency of co-occurrence or collaborative ties between two distinct nodes. “Total link strength” (TLS) is a cumulative metric calculated as the sum of link strengths of a given node over all other nodes in the network, representing its overall centrality. CiteSpace’s “burst strength” measures the intensity and rate of fluctuation of a keyword’s occurrence frequency within a specific timeframe using Kleinberg’s burst detection algorithm, which highlights rapid shifts in research focus.

### 2.3 Research ethics

Data were solely obtained from the publicly accessible WoSCC and PubMed databases. Since this study was a secondary bibliometric analysis and did not involve direct interaction with humans or animals, no ethical issues were present. Consequently, Ethics Committee approval, clinical trial registration, and informed consent were not required. This cross-sectional study has been conducted and reported in compliance with the STROBE (Strengthening the Reporting of Observational Studies in Epidemiology) guidelines (17).

## 3 Results

### 3.1 Literature screening and analysis of publication volume and yearly trends

The detailed literature selection process and the systematic screening results are illustrated in the PRISMA flow diagram (Figure 1). Mechanistically, the initial dual-database search retrieved a total of 1800 raw records (1,054 from the WoSCC and 746 from PubMed). After deduplication, which excluded 711 overlapping records, 1,089 unique articles remained. Applying the predefined eligibility criteria, we excluded non-peer-reviewed formats including meeting abstracts (*n* = 143), proceeding papers (*n* = 65), editorial materials (*n* = 31), letters (*n* = 13), early-access publications (*n* = 10), erratum/corrections (*n* = 9), meeting records (*n* = 1), and book chapters (*n* = 1). Nine non-English publications were also excluded. Consequently, a final refined cohort of 807 high-quality, peer-reviewed original research and review articles was established for subsequent bibliometric mapping.

In bibliometric research, traditional risk of bias assessment tools (such as Cochrane RoB or Newcastle-Ottawa scale) are not directly applicable since the unit of evaluation is the publication profile rather than clinical trial methodologies. To mitigate data bias and ensure data quality, we restricted our analysis to highly regulated peer-reviewed literature and controlled for publication bias by comparing visual output distributions with JCR journal impact factors, total local citations, and individual author/journal H-indices as proxies for methodological reliability and academic impact.

**Figure 2:**
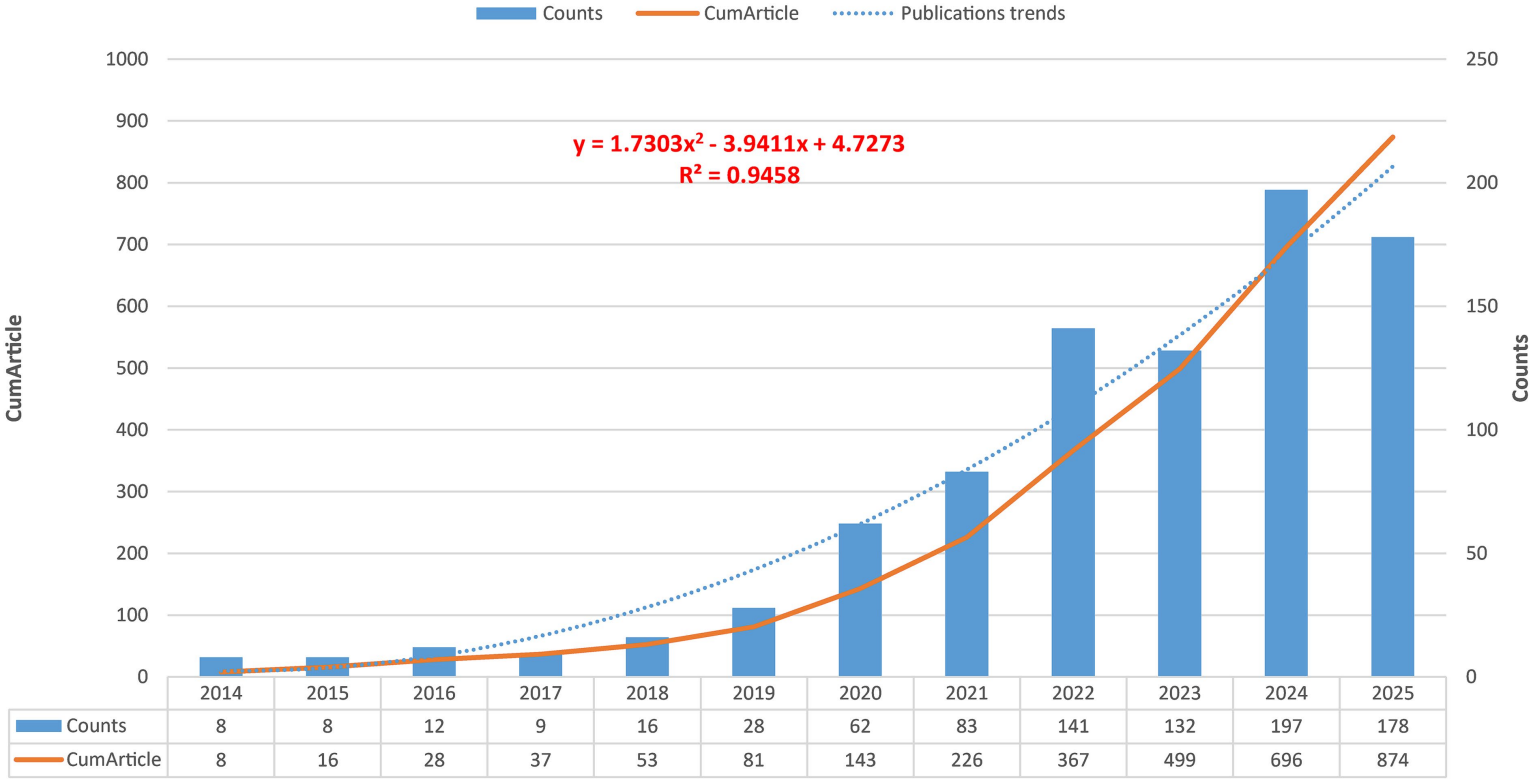
Annual publication output and cumulative growth trajectory of FLASH radiotherapy research (2014–2025). The primary *y*-axis (left) represents the annual publication count (blue bars), and the secondary *y*-axis (right) represents the cumulative volume of publications (orange line). The dashed blue line depicts the polynomial trend fitted to annual publication output, modeled by the quadratic equation *y* = 1.7303*x*^2^–3.9411*x* + 4.7273, with *R*^2^ = 0.9458, where *x* represents the chronological sequence of years starting from 2014. The data table aligned at the bottom provides exact annual counts.

The selected 807 publications span the full 12-year period from January 1, 2014, to December 31, 2025 (Figure 2). The field of FLASH-RT experienced a sustained escalation in scientific output, with a particularly pronounced surge beginning in 2021, which marks the clinical transition phase represented by the first prospective human trial. The annual global publication volume showed a rapidly accelerating, non-linear increase from 8 papers in 2014 to 178 in 2025, representing a 2,225% relative increase. The annual output reached its zenith in 2024 with 197 publications (24.4% of the total cohort). This growth pattern unequivocally marks a fundamental paradigm shift from exploratory preclinical proof-of-concept setups to standardized dosimetry verification and active phase-1 clinical workflows, defined by the standardization of dosimetry protocols and the transition to human trials.

**Figure 3:**
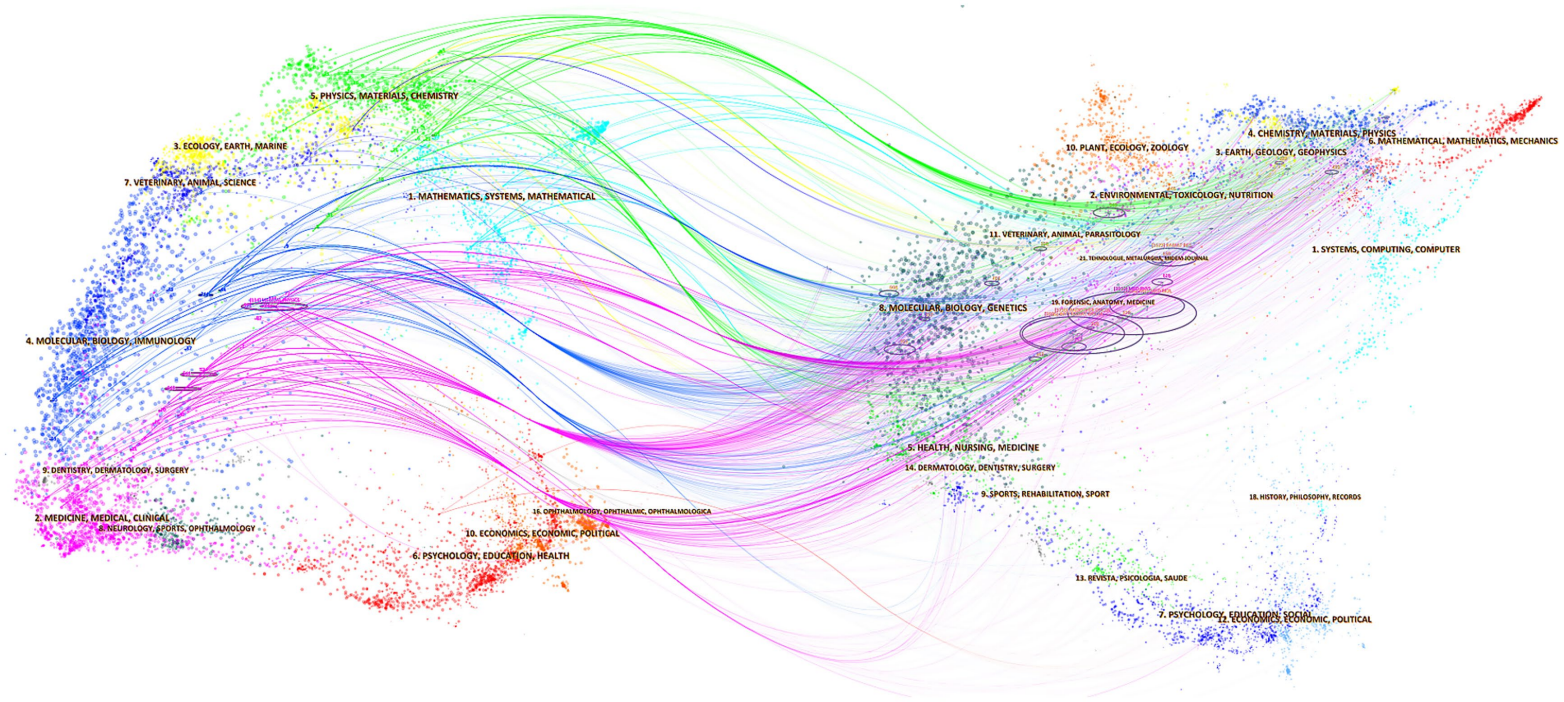
Dual-map overlay of citing and cited journals. This overlay visualizes the disciplinary citation mapping of FLASH-RT literature. Citing journals (source disciplines) are situated on the left, and cited journals (target disciplines) are situated on the right. Colored spline waves represent the paths of citation links, demonstrating the primary knowledge flows (e.g., from Physics/Materials/Chemistry and Medicine/Medical/Clinical to Health/Nursing/Medicine and Molecular/Biology/Genetics). Journal classifications are annotated in bold black text.

In Figure 2, the orange line represents the cumulative article count, whereas the dotted blue line denotes the fitted polynomial trend line for annual publication output. The fitted annual-output trend is described by the quadratic equation y = 1.7303x^2^–3.9411x + 4.7273, with R^2^ = 0.9458, where x represents the chronological sequence of years starting from 2014. Although the overall publication pattern reflects rapid acceleration, a polynomial trend line was selected rather than a purely exponential model because the observation window is finite, the annual output peaked in 2024 and slightly decreased in 2025, and a quadratic fit provides a conservative descriptive approximation without assuming indefinite exponential growth. Based on mathematical extrapolation of this historical trend, the model projects potential annual yields of 207 and 246 publications in 2026 and 2027, respectively. However, these values are mathematical estimations derived from trend extrapolation and are subject to database indexing delays and actual research fluctuations. The sustained average annual growth rate of 32.6% reflects the dynamic expansion of FLASH-RT research, while its interdisciplinary nature is further substantiated by citation pathway networks in the dual-map overlay (Figure 3) rather than annual growth metrics alone.

### 3.2 Distribution of countries/regions, institutions, and authors

The study analyzed publications from 53 different countries or regions. Table 1 lists the top 10 countries with the highest publication yield. The United States led quantitatively, accounting for the largest share of publications (*n* = 245, 30.4%) with 6,962 citations, followed by China (151 publications, 18.7%) and Italy (72 publications, 8.9%). Among European nations, Switzerland, Germany, France, and the United Kingdom ranked fourth, fifth, sixth, and eighth, respectively. Table 2 details the top 10 most productive institutions, with the University of Lausanne emerging as the leading global center (170 publications, 21.1%).

**Table 1:** The top 10 productive countries/regions.

Rank	Country/Region	Publications	Publications (%)	Citations
1	USA	245	30.4	6,962
2	China	151	18.7	1,549
3	Italy	72	8.9	1,240
4	Switzerland	57	7.1	3,842
5	Germany	48	5.9	1,577
6	France	43	5.3	2,312
7	Canada	41	5.1	619
8	United Kingdom	40	5.0	1,454
9	Korea	23	2.9	349
10	Japan	20	2.5	169

**Table 2:** The top 10 productive institutions.

Rank	Institution	Country	Total publications	Percentage (%)
1	University of Lausanne	Switzerland	170	21.1
2	Helmholtz Association	Germany	126	15.6
3	Centre Hospitalier Universitaire Vaudois	Switzerland	116	14.4
4	Stanford University	USA	102	12.6
5	Istituto Nazionale di Fisica Nucleare	Italy	100	12.4
6	Centres de Lutte Contre le Cancer, CLCC	France	93	11.5
7	Centre National de la Recherche Scientifique	France	85	10.5
8	Université PSL	France	78	9.7
9	Institut Curie	France	77	9.5
10	University of Pennsylvania	USA	74	9.2

Table 3 details the top 20 most prolific authors. MC Vozenin from the University Hospital of Geneva is the leading contributor with 45 publications and a total of 5,567 citations, averaging 124 citations per paper, primarily investigating the mechanism of the FLASH effect. The three authors with the highest overall cumulative citations were MC Vozenin, J Bourhis, and C Bailat. Notably, the works of J Bourhis from the University of Lausanne, particularly concerning proton-based FLASH-RT, exhibited the highest average citations, reaching 181 per publication.

**Table 3:** The top 20 productive authors.

Rank	Author	Country	Total publications	Total citations	Average citations	H-index	g-index
1	MC Vozenin	France/Switzerland	45	5,567	124	26	45
2	K Petersson	Switzerland	35	3,144	90	20	35
3	C Bailat	Switzerland	33	3,418	104	20	33
4	F Di Martino	Italy	26	370	14	11	19
5	F Bochud	Switzerland	24	2,704	113	17	24
6	J Bourhis	Switzerland	24	4,349	181	18	24
7	ML Kang	USA	24	440	18	12	20
8	HB Lin	USA	24	448	19	12	21
9	R Moeckli	Switzerland	23	2,211	96	17	23
10	Ji Choi	USA	22	410	19	11	20
11	G Felici	Italy	22	540	25	13	22
12	PG Jorge	Switzerland	21	2,448	117	17	21
13	MM Kim	USA	21	896	43	12	21
14	BW Pogue	USA	21	514	24	9	21
15	CL Limoli	USA	20	1,692	85	14	20
16	E Schüler	Germany	20	1,047	52	13	20
17	RX Zhang	USA	20	592	30	11	20
18	B Petit	Switzerland	19	2,494	131	16	19
19	V Grilj	Switzerland	18	692	38	12	18
20	BW Loo	USA	18	1,167	65	13	18

### 3.3 Co-authorship networks of countries/regions, institutions, and authors

**Figure 4:**
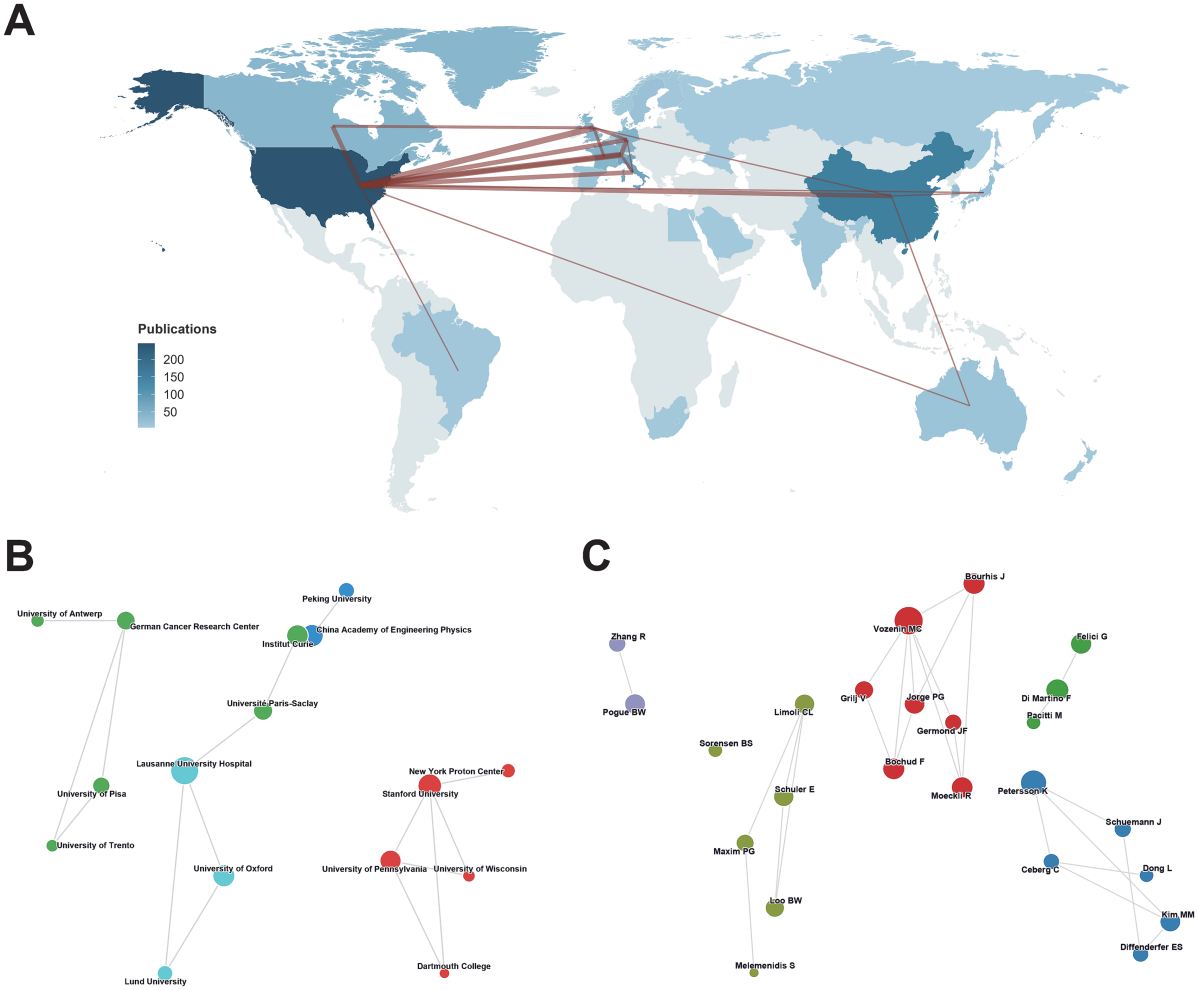
Global, institutional, and author collaborative networks in FLASH-RT research. **(A)** Country/Region Collaboration Map: Color opacity indicates publication volume (ranging from 50 or less to 200 or more publications), highlighting major contributions from the US and China. Red lines represent cross-national scientific collaborations. Taiwan is colored identically to mainland China to represent geographical integrity. **(B)** Institution Collaboration Network: Node size corresponds to the number of publications, and node colors denote distinct self-assembled collaborative clusters. **(C)** Author Collaboration Network: Nodes symbolize individual authors, with size reflecting their publication output. Colors characterize co-authorship research communities.

Co-authorship analysis at the country/region level revealed active global collaborative relationships (Figure 4A). The United States had the most cooperating partners (*n* = 33) and maintained robust connections with New Zealand, Japan, and Australia. The USA exhibited the highest total link strength (*n* = 171), with Switzerland and China closely following (*n* = 138 each). For visualizing the co-authorship network of institutions (Figure 4B), a minimum publication threshold of 10 articles per institution was set. The resulting network, encompassing 53 institutions, was delineated into 7 distinct clusters. The largest cluster (red), comprising 13 institutions, was notably centered around Stanford University. The second largest cluster (green) included 10 European universities, such as Université PSL, Università di Pisa, and University of Antwerp, which exhibited a decentralized structure. The third cluster (blue) consisted of 7 Chinese universities, including the China Academy of Engineering Physics, Peking University, and Sichuan University. Regarding total link strength among institutions, the University of Lausanne (*n* = 107) and the University of Oxford (*n* = 83) emerged as the top two.

The co-authorship network of individual authors was constructed based on a minimum threshold of 7 published articles and a total link strength of 2, resulting in 92 qualified authors divided into distinct color-coded collaborative communities (Figure 4C). Labels in Figure 4C are restricted to highly central authors to prevent visual overlapping. The largest collaborative group (red) is concentrated around Vozenin MC, Bourhis J, and Bochud F, focusing primarily on dosimetry and translation. Dr. F Di Martino, G Felici, and M Pacitti occupied a separate green cluster (Group 5), specializing in electron FLASH development and dosimetry. MC Vozenin had the highest overall linkage (*n* = 163) among all 4,017 authors, followed by C Bailat (*n* = 159) and F Bochud (*n* = 131).

### 3.4 Examination of source journals and citation metrics

A total of 222 distinct journals published literature within the FLASH-RT research field. Table 4 shows that the top 10 journals contributed to 51.9% of the total output. Medical Physics (IF 3.2) emerged as the most productive journal, contributing 114 publications, and also the most highly cited, amassing 3,889 citations. It was followed by Physics in Medicine & Biology (IF 3.5) with 72 publications, and Radiotherapy and Oncology (IF 5.3) with 52 publications.

**Table 4:** The top 10 journals published most on research on FLASH.

Rank	Source	Category	IF (2024)	Number of articles (percentage)	Total citations
1	Medical Physics	Medical Physics	3.2	114 (14.1)	3,889
2	Physics in Medicine and Biology	Medical Physics	3.5	72 (8.9)	2,255
3	Radiotherapy and Oncology	Oncology	5.3	52 (6.4)	3,705
4	International Journal of Radiation Oncology Biology Physics	Oncology	6.5	44 (5.5)	3,097
5	Frontiers in Oncology	Oncology	3.5	36 (4.5)	786
6	Cancers	Oncology	4.4	27 (3.3)	749
7	Frontiers in Physics	Physics	2.1	27 (3.3)	352
8	Radiation Research	Radiation Research	4.9	26 (3.2)	1,620
9	Physica Medica - European Journal of Medical Physics	Medical Physics	3.4	21 (2.6)	624
10	Scientific Reports	General Science	3.9	21 (2.6)	937

The 807 articles collectively received 20,142 citations, averaging 25.0 citations per article. Table 5 lists the 10 most locally cited publications. Of these top 10 papers, 7 focused on the radiobiological mechanisms of the FLASH effect (23–29), while the remaining 3 addressed clinical translation and implementation (30–32). The 2014 Science Translational Medicine article by Favaudon et al. was the top-ranked in both local and total citations (23). The pioneering clinical papers “The Advantage of FLASH Radiotherapy Confirmed in Mini-pig and Cat-cancer Patients” (24) and “Treatment of a First Patient with FLASH-radiotherapy” (30) ranked among the top three in total citations. Several leading first authors, such as V Favaudon, MC Vozenin, J Bourhis, and DR Spitz, demonstrated significant academic influence with individual h-index values exceeding 30.

**Table 5:** The top 10 highest locally cited articles.

Rank	Title (author)	Year	Journal	Local citations	Total citations (WoSCC)	H-index (first author)
1	Ultrahigh dose-rate FLASH irradiation increases the differential response between normal and tumor tissue in mice (Favaudon V et al)	2014	Science Translational Medicine	580	1,072	39
2	The Advantage of FLASH Radiotherapy Confirmed in Mini-pig and Cat-cancer Patients (Vozenin MC et al)	2019	Clinical Cancer Research	402	585	53
3	Treatment of a first patient with FLASH-radiotherapy (Bourhis J et al)	2019	Radiotherapy and Oncology	342	516	71
4	Long-term neurocognitive benefits of FLASH radiotherapy driven by reduced reactive oxygen species (Montay-Gruel P et al)	2019	Proceedings of the National Academy of Sciences of the United States of America	297	435	17
5	Biological Benefits of Ultra-high Dose Rate FLASH Radiotherapy: Sleeping Beauty Awoken (Vozenin MC et al)	2019	Clinical Oncology	242	385	53
6	Clinical translation of FLASH radiotherapy: Why and how? (Bourhis J et al)	2019	Radiotherapy and Oncology	237	373	71
7	Design, Implementation, and in Vivo Validation of a Novel Proton FLASH Radiation Therapy System (Diffenderfer ES et al)	2020	International Journal of Radiation Oncology*Biology*Physics	255	371	17
8	Ultra-High Dose Rate (FLASH) Radiotherapy: Silver Bullet or Fool’s Gold? (Wilson JD et al)	2020	Frontiers in Oncology	228	367	14
9	An integrated physico-chemical approach for explaining the differential impact of FLASH versus conventional dose rate irradiation on cancer and normal tissue responses (Spitz DR et al)	2019	Radiotherapy and Oncology	215	313	84
10	Hypofractionated FLASH-RT as an Effective Treatment against Glioblastoma that Reduces Neurocognitive Side Effects in Mice (Montay-Gruel P et al)	2021	Clinical Cancer Research	166	234	17

### 3.5 Analysis of keyword co-occurrence and detection of burst keywords

**Figure 5:**
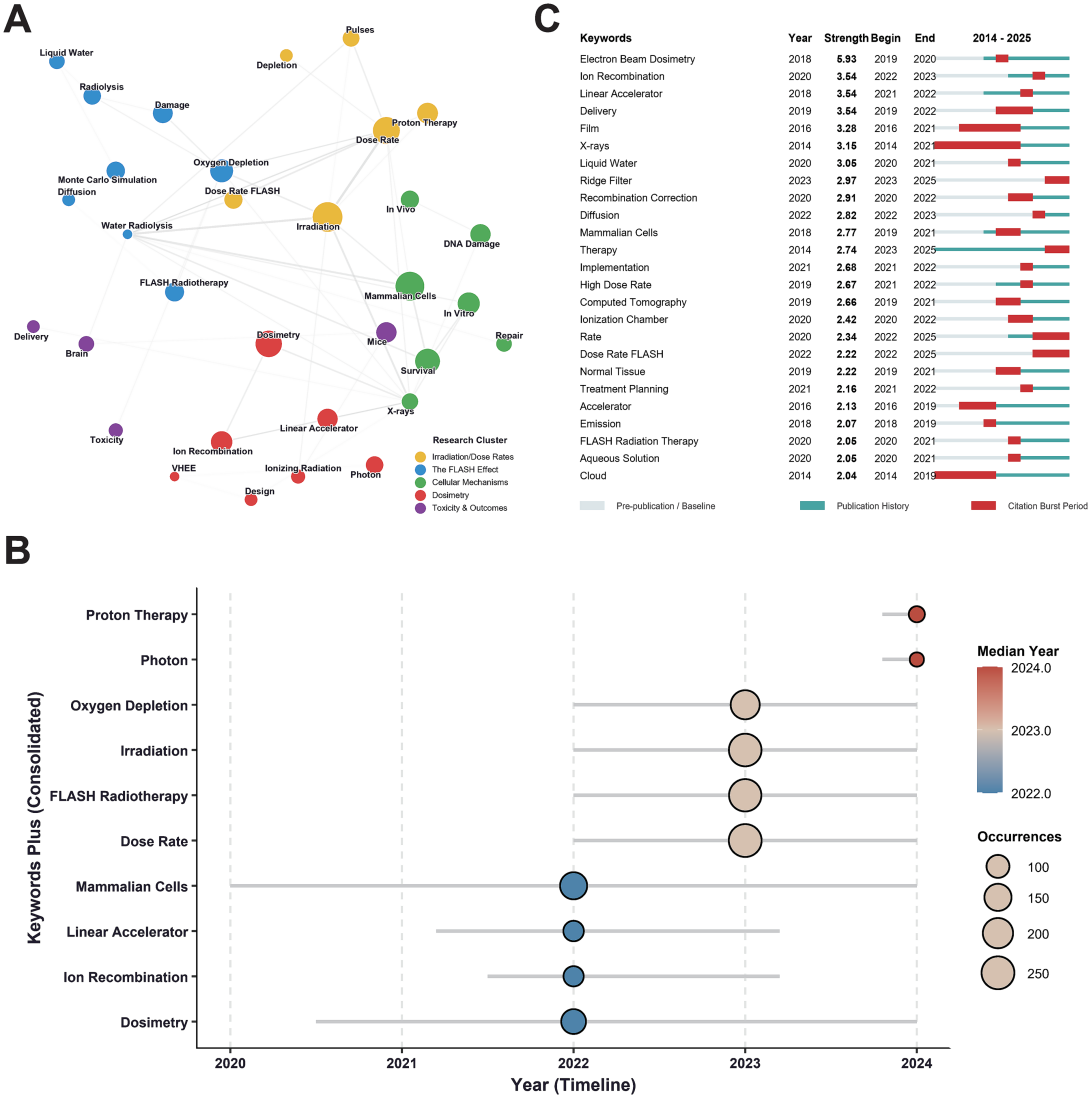
Keyword co-occurrence, timeline evolution, and citation bursts. **(A)** Keyword co-occurrence network: Node sizes indicate occurrence frequency. Nodes are partitioned into five research clusters: “Irradiation/Dose Rates” (yellow), “The FLASH Effect” (blue), “Cellular Mechanisms” (green), “Dosimetry” (red), and “Toxicity & Outcomes” (purple). **(B)** Keywords plus timeline view: Evolutionary trajectory of consolidated keywords, where horizontal bars span the active time range. Bubble sizes show the volume of occurrences, and the color gradient maps the median publication year from 2022 (blue) to 2024 (red). **(C)** Top 9 keywords with the strongest citation bursts: The red segments indicate the initiation, duration, and termination of active citation bursts, marking significant transitions in thematic interest from 2014 to 2025.

A total of 3,072 distinct keywords were identified. Using a minimum occurrence threshold of 10, a total of 104 high-frequency keywords were extracted, from which five primary clusters were established (Figure 5A): dosimetry (red), cellular mechanisms (green), the FLASH effect (blue), irradiation/dose rates (yellow), and toxicity/outcomes (purple). Cluster 1 (red) was characterized by keywords such as “dosimetry,” “ion recombination,” “beams” (label not visible in Figure 5A due to overlap), “ultra-high dose rate” (label not visible in Figure 5A due to overlap), and “linear accelerator.” Cluster 2 (green) centered on cellular responses, including “DNA damage,” “*in vitro*,” “X-rays,” “*in vivo*,” and “cancer” (label not visible in Figure 5A due to overlap). Cluster 3 (blue) featured “damage,” “FLASH effect” (label not visible in Figure 5A due to overlap), and “radiolysis.” Cluster 4 (yellow) consisted of “irradiation” and “dose rates.” Cluster 5 (purple) encompassed “toxicity” and “outcomes” (label not visible in Figure 5A due to overlap). Although related terms like “high dose rate,” “rate,” and “dose rate FLASH” describe similar physical concepts, they are mathematically separated to preserve their unique indexing and contextual usage in the databases.

To delineate the chronological evolution of these topics, a temporal overlay timeline was generated (Figure 5B). While studies focusing on “X-rays” and “mammalian cells” dominated the early stage, newer topics such as “proton therapy,” “oxygen depletion,” “delivery,” “rate,” and “dose rate FLASH” have thrived. The “dosimetry” cluster has experienced particularly rapid development, while studies concerning conventional “X-rays” appear less contemporary. This temporal pattern should not be interpreted as a decline in the importance of dosimetry itself; rather, it indicates a shift from broad early-stage electron-beam characterization toward more specific ultra-high-dose-rate challenges, including ion recombination, dose-rate specification, and delivery verification.

Figure 5C illustrates the top 9 burst keywords in this field from 2014 to 2025. The vertical line was added to emphasize the transition point associated with the onset of prospective human FLASH-RT trials and the subsequent clinical pivot in 2021. The statistical intensity of the citation surge is indicated by the “Strength” metric. The “Begin” and “End” columns map the active duration of the burst. “Electron beam dosimetry” exhibited the highest burst strength (5.93, active starting in 2019), followed by “ion recombination” (3.54, active key translation period 2022 to 2023), “linear accelerator” (3.54, active since 2021), and “delivery” (3.54, active from 2019 to 2022). More recent burst topics indicating current frontiers include “diffusion,” “rate,” and “dose rate FLASH.” Together, these burst keywords indicate that the field has moved from platform establishment toward clinically actionable issues of dose delivery, dose-rate definition, and treatment implementation.

### 3.6 Examination of influential publications

A stratified analysis was performed on the top 10% most cited articles (*n* = 81) to distinguish high-impact research (see Supplementary Table 1). The citation counts for these publications online varied between 63 and 1,072, with an average of 157 citations. The publications occurred in high-impact journals including JAMA Oncology (IF 20.1), Science Translational Medicine (IF 14.6), PNAS (IF 9.1), IJROBP (IF 6.5), Radiotherapy and Oncology (IF 5.3), and Annual Review of Cancer Biology (IF 6.1). Approximately half of these highly cited papers concentrated on physical beam configurations, specifically linear accelerators and oxygen depletion kinetics.

**Figure 6:**
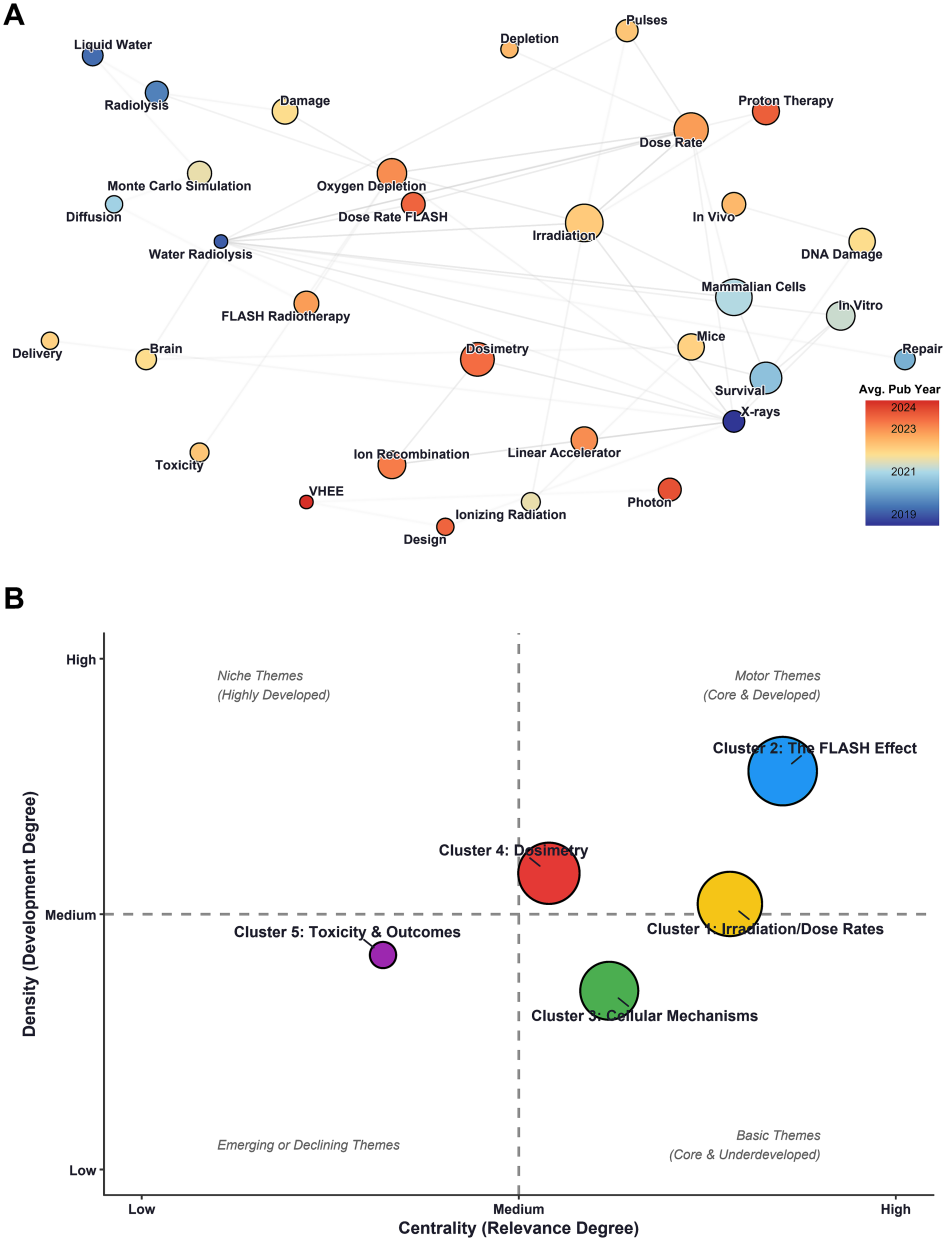
Chronological evolution and strategic thematic deployment of keywords. **(A)** Temporal keyword co-occurrence network: Nodes and paths are color-graded based on the average publication year of the keywords from 2019 (blue) to 2024 (red), visualizing the shift from early physical beam parameters to recent translational biology. **(B)** Thematic map (strategic quadrant analysis): Research clusters are mapped based on centrality (relevance degree, x-axis) and density (development degree, y-axis). Themes are classified into four quadrants: Motor themes (upper right), niche themes (upper left), emerging/declining themes (lower left), and basic/underdeveloped themes (lower right).

As depicted in Figure 6A, the temporal keyword network shows that the visible keyword “dosimetry” appears in the pink/red portion of the overlay, indicating that dosimetric research remains an active and relatively recent theme rather than only an early-stage topic. Therefore, we avoided attributing the early phase to “electron beam dosimetry,” which is not displayed as a visible label in Figure 6A. The more recent academic focus has transitioned toward clinically relevant and translational topics visible in the network, including “proton therapy,” “oxygen depletion,” and “delivery,” which appear closer to the red-orange/pink end of the temporal spectrum and demonstrate their current relevance. The strategic thematic map (Figure 6B) further shows that irradiation parameters, dose-rate-related themes, and translational delivery topics occupy different positions according to centrality and density, suggesting that the field is simultaneously consolidating core technical themes and developing newer clinically oriented research fronts.

The dual-map overlay (Figure 3) demonstrates that FLASH-RT is an inherently interdisciplinary field, bridging physical engineering, radiobiology, and clinical medicine. Specifically, the purple curves highlight significant citation structures flowing from Physics, Materials Science, and Chemistry to literature related to Physics and Molecular Biology. The green and orange curves depict major citation flows from Molecular Biology, Immunology, and Clinical Medicine to target journals of Molecular Biology & Genetics and Health, Nursing, & Medicine, highlighting the integration between fundamental physics and applied clinical oncology that drives the maturation of FLASH technology. Journal classifications on both sides are marked in bold black text for clarity.

## 4 Discussion

### 4.1 Global evolution: from physical verification to biology-driven clinical implementation

The inception of the modern FLASH era can be traced back to the seminal work of Favaudon et al. in 2014, which provided the first robust evidence of a differential dose–response between normal and tumor tissues under ultra-high dose rates (23). Since then, the 2,225% rapidly accelerating increase in publications identified in our dual-database study (Figure 2) signifies more than a mere increase in academic interest; it reflects a fundamental “paradigm shift” in radiation oncology. This trajectory indicates that FLASH-RT has successfully moved beyond an experimental curiosity into a stage of rigorous clinical validation.

A critical observation from our analysis is the maturation of research models. Between 2014 and 2020, research was predominantly physics- and radiobiology-heavy, focusing on the engineering of high-output accelerators—electron, photon, and proton beams—alongside basic dosimetric characterization and establishing basic toxicological profiles in small animal models (33–39). These preclinical studies were foundational, demonstrating that FLASH-RT could consistently mitigate late-stage side effects, such as pulmonary fibrosis and neurocognitive decline, without compromising tumor control (37–41). Our keyword burst analysis (Figure 5C) reveals a chronological pivot after 2021: as highlighted by the vertical reference line in Figure 5C, broad early technical terms such as “electron beam dosimetry” and “linear accelerator” were most prominent during the platform-establishment stage, whereas later burst terms such as “delivery,” “rate,” and “dose rate FLASH” reflect increasing attention to clinically implementable dose-rate specification and treatment workflow. Proton-related implementation is reflected more specifically by the visible keyword “Ridge filter,” rather than by a general “proton” burst keyword. Thus, the post-2021 transition should be interpreted as a shift from general accelerator and electron-beam characterization toward clinically oriented delivery optimization and proton-beam shaping strategies.

The leadership of the United States and Switzerland in this field is driven by distinct strategic architectures. The U.S. dominance stems from its broad institutional network and diverse funding through the NIH and private sectors, fostering a decentralized yet highly collaborative research ecosystem (Figure 4). This structural divergence is highly reflective of their respective funding landscapes: U.S. research is largely driven by collaborative multi-institutional National Institutes of Health (NIH) grants that favor consortium-based networks, whereas Swiss research has been underpinned by highly centralized institutional financing (such as the Bilateral and Swiss National Science Foundation schemes) that prioritizes technology-driven, localized expertise at hubs like the University of Lausanne and CHUV (30, 42).

The transition to human trials, notably the FAST-01 and FAST-02 studies (Table 6), represents a watershed moment (10, 12, 43). Early interim data from these trials confirm that proton FLASH-RT is not only safe but provides effective pain relief for bone metastases comparable to conventional treatments, yet with significantly reduced session times (9). Furthermore, the emerging focus on utilizing modified conventional clinical linear accelerators underscores the inherent synergy between FLASH technology and localized tumor control. By delivering high-dose-rate radiation, clinicians can potentially improve local control in complex tumors while minimizing late toxicity and exposure to adjacent critical organs—a clinical pathway that significantly expands the therapeutic window for patients (4, 44). Ultimately, the future deployment of this modality will center on the development of compact accelerators and targeted beams to enable deep-seated radical treatments (45–48).

**Table 6:** FLASH in phase 1 or 2 clinical trial.

Disease	Clinical trial	Study phase	Study focus	Group	Primary outcome measures	Conclusions	Ref.
Symptomatic Bone Metastases	FAST-01 (NCT04592887)	Phase 1	To assess the feasibility of FLASH radiotherapy for the palliative treatment of painful bone metastases.	Proton therapy Single-arm trial: FLASH-RT; 8Gy × 1 1–3 bone metastases in extremities 10 patients	Workflow Feasibility On-table time: >1 h (assessed on treatment day). System delay: >7 business days (simulation to treatment) due to device (assessed within 4 weeks enrollment). FLASH-RT Toxicities: Assessment: Treatment start to death/LOU. Measure: Possible/probable/definite FLASH-RT related toxicities (CTCAE v5.0).	Trial completed; Efficacy and safety of FLASH treatment was similar to CONV proton therapy	10
Symptomatic Bone Metastases in the Thorax	FAST-02 (NCT05524064)	Phase 1	To assess toxicities of FLASH radiotherapy treatment and pain relief in subjects with painful thoracic bone metastases	Proton therapy Single-arm trial; 8 Gy × 1 1–3 thorax bone metastases 10 patients (12 actually enrolled)	FLASH-RT Toxicities: Assessment: Treatment start to 6 months post-treatment. Measure: Possible/probable/definite FLASH-RT toxicities (CTCAE v5.0). Pain Relief (3 months): Overall Pain: Change from baseline (patient-reported). Treated Site Pain: Change from baseline (patient-reported). Pain Med Use: Change from baseline (dose/frequency).	June 2024 interim data: 8 Gy proton FLASH for 12 thoracic bone mets patients yielded 75% pain relief, no cardiorespiratory damage.	12
Skin Cancer	LANCE (NCT05724875)	Phase 2	FLASH vs. Conventional RT: Toxicity/Efficacy (Lesion Size) (Adding context about lesion size)	Electron therapy Arm 1: FLASH-RT; 22Gy × 1or5 × 6Gy Arm 2: CONV-RT; 22Gy × 1 or 5 × 6Gy T1-T2 NO cutaneous BCC or SCC 60 patients	Safety: Frequency of > = Grade 3 Skin Toxicity AEs (CTCAE v5.0) Efficacy: Local Control Rate (assessed via tumor response: CR, PR, PD, SD)	Recruiting	42
Malignant Melanomas	Flash-Skin I (NCT06549439)	Phase 1	Aims to assess feasibility and safety of electron Flash RT for treatment of melanoma skin metastases	RADIATION: Electron FLASH radiotherapy	Dose Limiting Toxicity (DLT) (Safety) [9 months] H0: > 2/6 patients in FLASH-irradiated lesions develop DLT. H1: ≤2/6 patients in FLASH-irradiated lesions develop DLT. Dose Accuracy (Feasibility) [9 months] H0: > 2/12 FLASH-RT fractions have dose deviation > ± 10%. H1: ≤ 2/12 FLASH-RT fractions have dose deviation > ± 10%.	Active, not recruiting	44
Melanoma in a Pulse	IMPulse (NCT04986696)	Phase 1	To evaluate dose escalation of single-dose FLASH therapy for locally progressive skin melanoma metastases resistant to systemic treatments.	Electron therapy Arm 1 (<30 cc): FLASH-RT; 22, 24, 26, 28, 30, 32, and 34 Gy × 1 Arm 2 (>30 and <100 cc): FLASH-RT: 22, 24, 26, 28, 30, 32, and 34 Gy × 1 Cutaneous melanoma metastases 46 patients	Determine MTD or RP2D for small and large volume skin metastases (Days 1–28). Evaluate acute safety (DLT) of high-dose-rate RT per dose level using NCI CTCAE v5.0 (4 weeks post-treatment).	To be determined	45

### 4.2 Cluster 1: dosimetry—the gateway to safely expanding the therapeutic window

When examining the hot topics within the red cluster (Cluster 1: Dosimetry) in Figure 5A, it becomes clear that dosimetry represents the primary technical bottleneck in the clinical translation of FLASH-RT. This interpretation does not contradict the observation in Section 4.1 that broad early technical labels are becoming less dominant. Rather, the bibliometric pattern indicates that the field has evolved from general electron-beam dosimetry and accelerator characterization toward more specific unresolved dosimetric problems, particularly ion recombination under ultra-high-dose-rate conditions. Traditional ionization-chamber-based dosimetry relies on ion collection; however, ionization chambers are highly susceptible to “ion recombination” effects under ultra-high dose rates, leading to significant signal saturation and measurement inaccuracies. This explains the explosive growth of keywords such as “ion recombination” in Figure 5C. Although “beam monitoring” is not displayed as an independent keyword in Figure 5A or Figure 5C, the need for online beam surveillance is conceptually linked to visible terms such as “dosimetry,” “linear accelerator,” and “delivery.” To address this core scientific issue, the field is pivoting toward dose-rate-independent systems, such as Faraday cups, radiochromic films, alanine dosimeters, and beam current transformers for clinical-grade real-time detection (49–53). The robustness of these tools is critical because any uncertainty in dose delivery at FLASH speeds could lead to catastrophic normal tissue injury or tumor under-dosing.

Our findings underscore that dosimetry in FLASH-RT is not merely about measuring quantity (Gy) but also about characterizing “spatio-temporal” parameters. As keywords like “pulse width” and “delivery methods” surge, it is clear that the FLASH effect is sensitive to the fine structure of the radiation delivery (54–57). For instance, in proton therapy, the transition to advanced pencil-beam scanning (PBS) requires precise optimization to ensure that every spot achieves the requisite dose rate while maintaining a uniform dose distribution across the target volume. Failure to account for the “inter-field” time intervals can dilute the effective dose rate, potentially “switching off” the biological FLASH effect (8, 58, 59).

This technical maturity is directly informing the design of pivotal clinical trials. The FAST-01 trial demonstrated that when dosimetric accuracy is rigorously maintained, clinical workflows are feasible, and outcomes are comparable to conventional therapy but with radically shortened delivery times (9, 10). Building on this, the FAST-02 trial has expanded its scope to thoracic metastases, where the dosimetric challenges are compounded by respiratory motion (12). The “bench-to-bedside” feedback loop is now reportable: the real-time dose measurement challenges encountered in FAST-01 are currently driving new preclinical research into real-time beam-current transformers and plastic scintillator detectors (51, 52).

However, significant hurdles remain. Establishing tissue-specific “injury thresholds” is mandatory, as the protective FLASH effect varies between organs such as the skin, bowel, and lung (33, 35, 60, 61). These statements are supported by the visible co-occurrence structure around “dosimetry,” “ion recombination,” “linear accelerator,” and “delivery,” which together indicate that accurate ultra-high-dose-rate measurement and controlled treatment delivery remain central translational requirements. Future research must prioritize the development of standardized “quality assurance” (QA) protocols that can validate ultra-high-dose-rate pulses in real-time. The transition from “experimental dosimetry” to “certified clinical metrology” will be the defining theme of the next era of FLASH research.

### 4.3 Cluster 3: the FLASH effect

While earlier investigations primarily focused on the selection of FLASH-RT devices and the accurate detection of dose thresholds, our keyword mapping in the blue cluster (Cluster 3: The FLASH Effect, Figure 5A) indicates a sustained and heightened interest in the biological determinants of the FLASH effect itself. The origins of the FLASH effect can be historically traced back to the 1970s, when seminal studies by Hornsey and Bewley first observed that high dose-rate irradiation possessed the capacity to reduce radiation damage to normal mouse tissues (62, 63). However, due to significant technical constraints in reliably achieving the requisite ultra-high dose rates, this groundbreaking finding did not garner broad attention. It was not until 2014 that the truly groundbreaking research by Favaudon et al. unequivocally demonstrated that ultra-high dose-rate irradiation could effectively inhibit tumor growth while significantly reducing adverse reactions in surrounding normal tissues (23, 64). This pivotal discovery effectively reignited scientific interest. This significant effect has been consistently validated in various animal models across multiple organs (7, 24, 65). Its applicability has also been successfully extended to different types of radiation particles, including X-rays and protons (66, 67). Additionally, the physical principles of flash radiography and high-yield X-ray pulses have historical applications in shock dispersal measurements and industrial image analysis (68, 69).

At its core, the FLASH effect resides in its remarkable capacity to significantly enhance the radiation tolerance of normal tissues. Research consistently shows that the protective effect becomes evident at dose rates over 30 Gy/s and is more pronounced above 100 Gy/s (70–72). Achieving a stable and reproducible FLASH effect is intricately linked to several critical parameters, which encompass physical attributes such as dose rate, absorbed dose, pulse width, and duty cycle, as well as physiological parameters like tissue oxygenation (11, 40, 70, 72). Precise dose monitoring under ultra-high dose-rate conditions remains a formidable challenge, as existing conventional dosimetry tools tend to exhibit saturation during such ultra-high dose-rate pulsed irradiations (73, 74). Currently, clinical films are widely employed for FLASH effect measurements due to their inherent dose-rate independent characteristics (52, 75). Ultimately, while FLASH-RT’s millisecond-level delivery rates hold immense potential to mitigate uncertainties stemming from organ motion in moving structures (e.g., lungs), integrating tumor tracking remains indispensable to ensure that the target volume continues to receive the narrow beam profile without spatial deviation (76).

### 4.4 Cluster 2: cellular mechanisms

Within this research domain (represented by the green cluster, Cluster 2: Cellular Mechanisms, in Figure 5A), the observed shift in prominent keywords from “X-rays” to “oxygen depletion” over the past 12 years strongly reflects a fundamental transition in the exploration of the FLASH effect’s underlying mechanisms. This shift aligns with the transition from simple radiochemical free-radical kinetics in pure water (traditionally studied using conventional X-rays) to the complex physiological dynamics of intracellular dissolved oxygen depletion, which varies drastically between normal and hypoxic tumor microenvironments. Early investigations attempted to elucidate the FLASH effect from a purely radiochemical perspective, primarily focusing on alterations in free radical generation (such as reactive oxygen species, ROS) under ultra-high dose rate irradiation (5). However, experimental results derived from cell-free systems yielded inconsistent findings. Even in instances where reduced hydrogen peroxide production or decreased lipid-peptide oxidative damage was observed under FLASH conditions (77, 78), such physicochemical changes, theoretically, should confer equal protection to both tumor and normal tissues (79). This premise contradicts the empirically observed selective protection of normal tissues *in vivo*, strongly indicating that biological systems themselves are the pivotal determinants of the FLASH effect (80).

Further studies have consistently demonstrated that FLASH-RT markedly raises the dose threshold for normal tissue toxicity in both early-responding tissues and late-responding tissues, with a significantly stronger protective effect on late-responding tissues (60, 81). Transcriptomic analysis has further revealed that, in comparison to conventional radiotherapy, FLASH-RT resulted in a lower upregulation of genes critically related to DNA damage response, inflammatory response, and profibrotic processes across various normal tissues (25, 82, 83). Conversely, pathways associated with tissue repair, angiogenesis, and specific differentiation were notably better maintained. In a compelling zebrafish embryo model, FLASH-RT demonstrably preserved the embryo’s intrinsic repair and morphogenetic potential (82). These aggregated results suggest that FLASH-RT triggers distinct biological response programs, thereby more effectively maintaining tissue homeostatic functions.

In stark contrast to normal tissues, the majority of preclinical studies consistently indicate that FLASH radiotherapy and conventional radiotherapy achieve remarkably similar cytotoxic effects on solid tumors at equivalent physical doses (6, 84–87). Classical radiobiological “5R” theory (including repair, reoxygenation, redistribution, repopulation, and intrinsic radiosensitivity) (28, 55), and even the emerging “6R” framework which incorporates the reactivation of the anti-tumor immune response (55), exhibit inherent limitations in adequately explaining this observed differential effect between normal and tumor tissues. While the lack of clear radiobiological models is frequently highlighted as a major academic hurdle, it may not prevent clinical adoption once sufficient evidence from clinical trials establishes safety and non-inferiority. Nevertheless, a robust radiobiological model remains essential for prospective patient stratification and optimal dose programming.

### 4.5 Challenges encountered in FLASH-RT research

Despite the extensive potential and promising outlook presented by preclinical FLASH-RT research, several formidable technical, safety-related, and methodological challenges must be meticulously addressed. One primary challenge lies in the precise setting and real-time measurement of the dose rate. FLASH-RT confronts significant issues in accurately determining and continuously monitoring precise doses, particularly when employing ultra-high dose rate beams (6, 66, 88).

Secondly, a persistent lack of clear biological mechanisms and robust research models remains a substantial hurdle. A more comprehensive and precise comprehension of the FLASH effect’s underlying mechanisms would unequivocally greatly facilitate its safe and effective clinical application (3, 11, 89). Furthermore, the development and optimization of advanced radiotherapy sources present considerable hurdles. Finally, larger-scale, meticulously designed clinical trials are urgently required to systematically compare FLASH-RT with conventional radiotherapy techniques. Such extensive evaluation will be indispensable in providing a robust and reliable basis for its widespread clinical adoption.

### 4.6 Future perspectives

Future research directions must prioritize a deeper elucidation of the precise biological mechanisms underpinning the FLASH effect. This includes initiating targeted clinical trials specifically designed to investigate the cellular and molecular pathways that confer normal tissue sparing and maintain tumor control. This innovative technique is particularly effective for tumors that are poorly responsive to conventional radiotherapy. Furthermore, FLASH-RT can be effectively integrated with other therapeutic modalities to significantly enhance the overall efficacy of cancer treatment (83, 90).

Looking ahead, FLASH-RT’s ultra-fast delivery reduces uncertainties from organ motion in tumors located in moving organs like the lung and gastrointestinal tract, enhancing treatment precision (76). Moreover, its sequential/concurrent application with chemotherapy or immunotherapy is anticipated to further enhance overall tumor treatment efficacy and patient survival rates (16). Moving forward, the innovation of compact, high-power FLASH accelerators is expected to furnish more flexible treatment options and broader clinical accessibility (91). Concurrently, the refinement of dosimetry measurement and real-time monitoring systems is anticipated to surmount the saturation limitations, thus ensuring the precision and safety of FLASH treatments (92).

### 4.7 Limitations

Although this bibliometric analysis provides a quantitative and objective mapping of the FLASH-RT landscape, several inherent limitations must be acknowledged. First, while we proudly implemented a dual-database integration strategy utilizing both the Web of Science Core Collection (WoSCC) and PubMed to ensure maximum comprehensiveness across physical sciences and biomedical translation, we may still have omitted relevant studies indexed exclusively in other databases (such as Scopus) and “grey literature” like conference proceedings. Second, bibliometric analysis treats all included publications with equal weighting based on citation counts. Citation metrics are naturally retrospective and subject to “time-lag”; consequently, groundbreaking studies published recently may not yet reflect their true academic impact. Third, our search was restricted to English-language articles, which might introduce a geographic or linguistic bias. Lastly, a bibliometric overview is primarily descriptive and relies on mapping “trends and patterns,” which does not replace the need for a rigorous systematic review or meta-analysis. However, by identifying the critical “Research Bursts” across these two major interconnected databases, this study provides a robust, evidence-based framework for tracking the evolution of FLASH-RT.

## 5 Conclusion

This study provides a comprehensive bibliometric roadmap of the FLASH radiotherapy landscape from 2014 to 2025, capturing a pivotal decadal transition from “laboratory curiosity” to “clinical reality.” By systematically integrating high-quality data from both WoSCC and PubMed, our findings illustrate an unprecedented acceleration in research output. Our visualized analysis identifies a definitive paradigm shift: early-stage investigations centered on physics-based beam monitoring and hardware engineering have now converged deeply with molecular biology and clinical oncology protocols.

The three primary research clusters—dosimetry, cellular mechanisms, and the FLASH effect—are deeply interlinked to facilitate the “clinical pivot.” For the broader oncology community, FLASH technology presents a transformative opportunity. By delivering ultra-high doses in milliseconds, FLASH-RT effectively mitigates uncertainties from internal organ motion, maximizing tumor ablation while preserving the integrity of adjacent critical organs. Looking ahead, the scalable success of FLASH research will hinge on standardizing real-time clinical dosimetry to overcome ion recombination effects and deeper elucidation of biological mechanisms. Ultimately, FLASH-RT is poised to widen the therapeutic window in oncology, offering a disruptive precision tool that harmonizes innovations in physics, biology, and clinical medicine.

## Data Availability

The original contributions presented in the study are included in the article/Supplementary material, further inquiries can be directed to the corresponding author.
